# Audit of the Management of High Clozapine Levels Within Solihull Community Mental Health Teams (CMHTs)

**DOI:** 10.1192/bjo.2024.609

**Published:** 2024-08-01

**Authors:** Sarah O'Connor, Huma Hashmat

**Affiliations:** Birmingham and Solihull Mental Health Foundation Trust, Birmingham, United Kingdom

## Abstract

**Aims:**

To review the practice of management of clozapine plasma levels in Solihull CMHTs between April and September 2023. Birmingham and Solihull Mental Health Foundation Trust (BSMHFT) clozapine guidelines were issued in January 2023 and further ratified in December 2023. The standard set out in the January 2023 guideline was that service users with elevated clozapine levels >600mcg/L should be assessed for signs of toxicity and consideration given to a dose reduction. Those with levels above 1000mcg/L should be reviewed urgently.

**Methods:**

Clozapine blood clinic diaries were reviewed in order to obtain a list of 48 service users who had attended for clozapine blood tests between April and September 2023. Blood results were reviewed for clozapine level results. For those service users whose clozapine level had been over 600mcg/L, clinical notes were reviewed to determine whether they had been screened for clozapine toxicity.

**Results:**

Of the 48 service users prescribed clozapine, 24 had clozapine levels over 600mcg/L and 11 had levels over 1000mcg/L. Of the service users with clozapine levels over 600mcg/L, 16 (67%) were screened for toxicity. Of those with clozapine levels over 1000mcg/L, 9 (82%) were screened for toxicity.

**Conclusion:**

Between April and September 2023, Solihull CMHTs demonstrated an understanding of the need for actioning elevated plasma levels as a priority, however, this could be further improved. The risks of adverse effects and toxicity with clozapine increase with raised plasma levels, particularly with levels over 1000mcg/L. Therefore, it is important that raised plasma levels are actioned accordingly. Locally, we have implemented a flow chart which summarises the updated clozapine guidelines, to assist clinicians in interpreting and acting on high clozapine levels and to prompt clinicians to review service users for signs of toxicity. We hope to incorporate this visual aid into the updated BSMHFT guidelines.

